# Cytomegalovirus Infection Is Associated with Accelerated Murine Glioblastoma Progression and Alterations in the Tumor Microenvironment

**DOI:** 10.3390/cells15211957

**Published:** 2026-10-28

**Authors:** Mykola Zdioruk, Eleni Panagioti, Noe B. Mercado, Vincent van Unen, Ennio Antonio Chiocca, Michal O. Nowicki, James A. Lederer, Sean E. Lawler, Charles H. Cook

**Affiliations:** 1 Harvey Cushing Neuro-Oncology Laboratories, Department of Neurosurgery, Mass General Brigham Neuroscience Institute, Boston, MA 02115, USA; myko.zdioruk@gmail.com (M.Z.);; 2 Harvard Medical School, Boston, MA 02115, USA; epanagio@bidmc.harvard.edu (E.P.);; 3 Department of Surgery, Beth Israel Deaconess Medical Center, Boston, MA 02215, USA; 4 Department of Pathology and Laboratory Medicine, Legorreta Cancer Center, Brown University, Providence, RI 02903, USA; 5 Department of Immunology, Leiden University Medical Center, 2300 RC Leiden, The Netherlands; 6 Center for Tumors of the Nervous System, Mass General Brigham Cancer Institute, Boston, MA 02114, USA; 7 Department of Surgery, Mass General Brigham, Boston, MA 02115, USA

**Keywords:** cytomegalovirus, glioblastoma, vascularization, tumor microenvironment

## Abstract

**Highlights:**

**What are the main findings?**

- Pre-existing mCMV infection is associated with accelerated GBM progression across four genetically distinct murine glioma models.
- mCMV-associated GBM progression is accompanied by vascular and immune remodeling of the tumor microenvironment.

**What are the implications of the main findings?**

- CMV-associated remodeling of the tumor microenvironment may represent a potential therapeutic vulnerability in GBM.
- These findings support further investigation of CMV-directed strategies targeting the GBM microenvironment.

**Abstract:**

Glioblastoma (GBM) is a highly aggressive brain tumor with no effective treatments. It is characterized by rapid progression and marked heterogeneity, which contributes to therapeutic resistance. Human cytomegalovirus (hCMV), a highly prevalent β-herpesvirus, has been associated with GBM in numerous studies, and both antiviral drugs and hCMV-targeted immunotherapies have shown evidence of therapeutic activity in clinical trials. However, the mechanisms underlying this association remain poorly understood. To address this, we previously developed a murine model of GBM with murine CMV (mCMV) infection, which revealed accelerated tumor progression in infected mice. Here, we investigated whether tumor heterogeneity influences the effects of mCMV infection on intracranial tumor growth across four murine GBM models (GL261, CT-2A, M005 and Mut3). Tumor progression was monitored longitudinally by MRI and evaluated by survival analysis, histopathology and characterization of the tumor microenvironment. Systemic mCMV infection was associated with accelerated GBM progression across all models, characterized by increased tumor growth and significantly reduced survival. Enhanced tumor progression was accompanied by increased vascularization, elevated myeloid cell activation and intratumoral viral activity in a subset of tumors. Mass cytometry (CyTOF) revealed remodeling of the tumor immune landscape, including a marked increase in intratumoral virus-specific CD8^+^ T cells, highlighting a potential avenue for therapeutic targeting. Together, these findings indicate that CMV is associated with accelerated GBM progression across genetically distinct murine models, accompanied by remodeling of the tumor microenvironment, including increased vascularization and altered immune infiltration.

## 1. Introduction

Glioblastoma (GBM) is the most common primary malignant brain tumor in adults [1,2]. GBM is highly heterogeneous at both cellular and molecular levels, which is a major contributor to therapeutic resistance [3,4]. This genetic diversity, together with an aggressive and invasive phenotype, makes GBM exceptionally difficult to treat [5]. Even with standard-of-care therapy, surgery, radiation, and temozolomide [5,6,7], patients have a median survival of only 15 months [8], underscoring the urgent need for more effective treatments [5].

Recent evidence has suggested a link between human cytomegalovirus (hCMV) and GBM progression. hCMV is a β-herpesvirus that infects a large percentage of the population [9], and after initial acute infection is controlled, it becomes dormant in its host. Like other members of the herpesviridae family, hCMV can reactivate under certain conditions, such as immunosuppression, and cause pathology. Interestingly, hCMV seropositivity has been linked to poorer prognosis in GBM patients [10], and numerous groups have detected hCMV proteins and nucleic acids in human tumor tissue specimens [11,12,13,14,15,16,17,18,19,20,21,22,23,24,25,26,27,28,29,30,31,32,33,34,35]. Moreover, several clinical trials targeting hCMV have shown promising responses in GBM patients. For example, autologous dendritic cell vaccines expressing the hCMV immunodominant antigen pp65 were associated with 30% long-term survival (>5 years) in recurrent GBM [36,37,38,39,40], and adjuvant valganciclovir treatment has been linked to significantly prolonged survival in newly diagnosed GBM (median 24.1 months) [41,42,43]. Despite these observations, the mechanistic relationship between CMV and GBM progression remains poorly understood.

To address this question, we previously developed an in vivo murine model combining CMV infection and GBM. In this model, mice are infected with murine CMV (mCMV) during the perinatal period, and GBM cells are implanted 16 weeks later, when viral activity is no longer detectable. Using this model, we found that prior mCMV infection was associated with accelerated GBM progression in vivo, with mCMV-infected mice exhibiting significantly shorter survival, increased tumor growth and enhanced vascularization in the GL261 model [44,45,46]. Here, we extend these findings by evaluating the effects of mCMV infection on GBM progression across multiple murine models, thereby capturing aspects of tumor heterogeneity and determining whether the association between pre-existing mCMV infection and tumor progression is reproducible across genetically distinct GBM models. We further assessed the susceptibility of each GBM cell line to viral infection and characterized the associated changes in the tumor microenvironment. CMV infection is associated with profound remodeling of the tumor microenvironment, including increased vascularization, altered immune infiltration, activation of resident myeloid cells and sporadic viral activity in a subset of tumors. These findings provide new insights into the potential relationship between pre-existing CMV infection and tumor progression and support further investigation of CMV as a therapeutic target in GBM.

## 2. Materials and Methods

### 2.1. Study Design

The aim of this study was to characterize peritumoral and tumor microenvironmental changes associated with mCMV infection during GBM progression. The impact of mCMV on GBM progression was assessed using a mCMV-GBM model incorporating four distinct glioma cell lines. Sample sizes for animal experiments (n = 3–6) were based on previous experiments using these models [45]. Mouse survival and tumor burden were the primary outcomes. Biological replicates are indicated by *n* in the corresponding figure legends.

### 2.2. Cell Culture and Virus Propagation

NIH3T3 mouse fibroblast cells were purchased from ATCC (ATCC, catalog number CRL-1658) and cultured in DMEM (Thermo Fisher Scientific, Waltham, MA, USA, catalog number 11965092) supplemented with 10% FBS (Sigma-Aldrich, St. Louis, MO, USA, catalog number F4135) and penicillin/streptomycin (100 μg/mL) (Thermo Fisher Scientific, catalog number 15140122). mCMV expressing GFP (Smith strain background) [47] was a kind gift from the laboratory of Dr. Michael Brown (University of Virginia School of Medicine, Charlottesville, VA, USA). mCMV was propagated and titrated in NIH3T3 cells, and viral titers were determined as previously described [45]. GL261-Luc2 murine glioma cells were purchased from PerkinElmer (Waltham, MA, USA), and CT-2A murine glioma cells were a gift from Dr. Thomas Seyfried (Boston College, Boston, MA, USA). Mut3 and M005 murine glioma cells were maintained in the laboratory. All four glioma cell lines were maintained as neurospheres in a 1:1 mixture of Neurobasal medium (Thermo Fisher Scientific, catalog number 21103049) supplemented with B27 (Thermo Fisher Scientific, catalog number 17504044), N2 (Thermo Fisher Scientific, catalog number 17502048), human EGF (20 ng/mL), human FGF (20 ng/mL), and 1% penicillin/streptomycin (Thermo Fisher Scientific, catalog number 15140122). Cells were cultured at 37 °C in a humidified atmosphere containing 5% CO_2_. Neurospheres were routinely dissociated and passaged to maintain proliferative cultures. Mycoplasma contamination was routinely assessed by PCR.

### 2.3. CMV Infection and Infectivity Test In Vitro

For in vitro mCMV infection, 2 × 10^3^ cells from each cell line (GL261Luc2, CT2A, M005 and Mut3) were seeded in 96-well ultra-low-attachment plates (Corning, New York, NY, USA, catalog number 7007), with eight technical replicates per cell line, and infected with GFP-labeled mCMV (mCMV-GFP) at a multiplicity of infection (MOI) of 1. Cells were imaged hourly for 96 h using a Nikon TE2000 fluorescence time-lapse microscopy system (Nikon Instruments Inc, Melville, NY, USA).

### 2.4. qRT-PCR

Total RNA and DNA were extracted using the AllPrep DNA/RNA/miRNA Universal Kit (QIAGEN, Venlo, The Netherlands, catalog number 80224, catalog number 80224). Nucleic acid detection and quantification were performed using the Luna Universal One-Step RT-qPCR Kit (NEB, Ipswich, MA, USA, catalog number E3005). RNA and DNA concentrations were quantified using a NanoDrop spectrophotometer (Thermo Fisher Scientific), and RT-qPCR was performed using an Applied Biosystems StepOnePlus Real-Time PCR System (Thermo Fisher Scientific). For detection of mCMV glycoprotein B (gB; mCMV Smith strain, GenBank accession no. GU305914.1), the following primers and probe were used: gB-CHC-for2, 5′-GAGAACTGCGACACGAACAG-3′; gB-CHC-rev2, 5′-AGCACCTTGAAGTCGGTGTT-3′; and the FAM-labeled hydrolysis probe gB-CHC-P1, 5′-[6-FAM]CGATGTCCAGCCCGATCAGG [BHQ-1]-3′. The gB-CHC-P1 probe was labeled at the 5′ end with 6-FAM and at the 3′ end with a Black Hole Quencher-1 (BHQ-1).

### 2.5. mCMV Model and In Vivo Studies

Six-week-old male and female C57BL/6 mice were purchased from The Jackson Laboratory and mated once. Female F1 mice were used for all subsequent experiments. The F1 generation was inoculated with 10^3^ PFU Δ157 mCMV at P2 by i.p. injection, as previously described [45]. Adult mice were infected with 10^5^ PFU by intraperitoneal (i.p.) injection and randomized before the start of the experiments. After 16 weeks, 5000 cells (GL261Luc2, CT2A, M005, Mut3) in 3 μL normal saline were injected intracranially to establish mouse brain tumors. Stereotactic tumor cell implantation was performed using the following coordinates: 2 mm right lateral, 1 mm frontal to the bregma, and 3 mm deep. CMV infection was confirmed by a serum anti-mCMV Multiplexed Fluorometric ImmunoAssay (Charles River Laboratories, Wilmington, MA, USA).

### 2.6. Imaging Methods

MRI data was acquired 20 days after tumor implantation using a 7-Tesla Bruker scanner (Bruker BioSpin, Billerica, MA, USA). Animals were maintained under isoflurane anesthesia throughout imaging. Respiration and heart rate were monitored, and body temperature was maintained using a homeothermic blanket. T2-weighted images were acquired using the rapid acquisition with relaxation enhancement (RARE) sequence. Tumor segmentations were manually reviewed for accuracy, and measurements were performed by the same investigators using predefined anatomical criteria. Investigators were not blinded to experimental group assignments.

### 2.7. Immunohistochemistry

Mice were euthanized using CO_2_ inhalation and subsequently perfused with 4% neutral-buffered formalin (Sigma-Aldrich, catalog number 100496) for fixation. Cryoprotection was performed using 30% sucrose. All mouse brain slides were obtained from 30 μm frozen sections. Slides were incubated with primary antibody (1:50 in normal serum) overnight at 4 °C. For primary antibody detection, species-matched fluorophore-coupled antibodies were incubated for 1 h at room temperature. Slides were then covered with anti-fade mounting medium (Vectashield, Vector Laboratories, Newark, CA, USA, catalog number H-1000-10) and cover slipped. All fluorescent and bright-field microscopy-based assays were observed using a Nikon Eclipse Ti microscope (Nikon Instruments, Melville, NY, USA). High-resolution confocal fluorescent microscopy was performed using a Zeiss LSM 710 confocal microscope system (Carl Zeiss Microscopy GmbH, Baden-Württemberg, Germany) and visualized using ZEN 2.1 (black, Zeiss) Imaging software. For colocalization, images were imported into the Fiji version of the free image-processing software ImageJ (https://imagej.net, NIH, Bethesda, MD, USA). For immunofluorescence studies, the following antibodies were used: anti-mouse, CD31 (Thermo Fisher, catalog number TLD-3A12, 1:50), anti-GFAP (Cell Signaling Technologies, Danvers, MA, USA, catalog number 80788, 1:150) anti-m123/IE1 (clone CROMA101, Center for Proteomics University of Rijeka, Rijeka, Croatia, catalog number HR-mCMV-08, 1:50) and anti-m57 mCMV (clone M57.02, catalog number -HR-mCMV-06, 1:50), and anti-Iba1 (Abcam, Waltham, MA, USA, catalog number ab5076, 1:250). The investigator performing the histological analyses was not blinded to the experimental groups.

### 2.8. Mass Cytometry Time of Flight (CyToF) Analysis of Tumor Immune Infiltrates

Exploratory CyTOF analysis was performed to characterize the immune infiltrate in tumors from mCMV-infected and mock-injected mice. Cryopreserved tumor-infiltrating lymphocyte (TIL) samples were thawed in a 37 °C water bath for 3 min and then mixed with 37 °C thawing media containing RPMI Medium 1640 (Life Technologies, Carlsbad, CA, USA, catalog number 11875-085) supplemented with 5% heat-inactivated fetal bovine serum (Life Technologies, catalog number 16000044), 1 mM GlutaMAX (Life Technologies, catalog number 35050079), antibiotic-antimycotic (Life Technologies, catalog number 15240062), 2 mM MEM non-essential amino acids (Life Technologies, catalog number 11140050), 10 mM HEPES (Life Technologies, catalog number 15630080), 2.5 × 10^−5^ M 2-mercaptoethanol (Sigma-Aldrich, catalog number M3148), 20 units/mL sodium heparin (Sigma-Aldrich, catalog number H3393), and 25 units/mL benzonase nuclease (Sigma-Aldrich, catalog number E1014). A 100 μL aliquot of each sample post-thaw was mixed with PBS (Life Technologies, catalog number 10010023) at a 1:1 ratio for cell counting by flow cytometry. Between 0.5 × 10^6^ and 1.0 × 10^6^ cells were used per sample. All samples were transferred to a polypropylene plate and stained at room temperature for the remainder of the experiment. The samples were centrifuged and aspirated. Cisplatin viability staining reagent (5 μM; Fluidigm, catalog number 201064) was added for 2 min and then diluted with culture media. Samples were fixed with 0.2% formaldehyde for 5 min using 16% stock paraformaldehyde (Fisher Scientific, catalog number O4042-500) diluted in PBS. After centrifugation (5810R, Eppendorf, Framingham, MA, USA), mouse anti-CD16/32 antibody Fc receptor blocking reagent (BioLegend, San Diego, CA, USA, catalog number 101302) was used at a 1:100 dilution in CSB (PBS with 2.5 g bovine serum albumin [Sigma-Aldrich, catalog number A3059] and 100 mg sodium azide [Sigma-Aldrich, catalog number 71289]) for 15 min, followed by incubation with conjugated surface antibodies for 30 min. All antibodies were obtained from the Harvard Medical Area CyTOF Antibody Resource and Core (Boston, MA, USA). Samples were subsequently fixed with 4% formaldehyde for 10 min and permeabilized using the FoxP3/Transcription Factor Staining Buffer Set (Thermo Fisher Scientific, catalog number 00-5523-00). Samples were incubated with SCN–EDTA-coupled palladium-based barcoding reagents for 15 min and then combined into a single sample. Following incubation in 100 units/mL heparin in PBS for 15 min, conjugated intracellular antibodies were added and incubated for 60 min. Samples were then fixed with 4% formaldehyde for 10 min. DNA was labeled for 20 min with an 18.75 μM iridium intercalator solution (Fluidigm, Boston, MA, USA, catalog number 201192B). Samples were washed and reconstituted in Maxpar Cell Acquisition Solution (Fluidigm, catalog number 201241) containing EQ Four Element Calibration beads (Fluidigm, catalog number 201078) at a final concentration of 1 × 10^6^ cells/mL.

Samples were acquired on a Helios CyTOF Mass Cytometer (Fluidigm). The raw FCS files were normalized to reduce signal deviation between samples over the course of the acquisition time, utilizing the bead standard normalization method established by Finck et al. [48]. Normalized files were compensated using a panel-specific spillover matrix and the CyTOF-based compensation method [49], followed by deconvolution into individual sample files using a single-cell-based debarcoding algorithm Files were gated to remove normalization beads and select live singlets in FlowJo. Live singlets were imported into Cytosplore (www.cytosplore.org) and embedded by t-SNE. Cell clusters were identified by density-based clustering on the t-SNE map and annotated based on marker-expression profiles. Statistically different clusters between groups were identified using the multiclass setting of Significance Analysis of Microarrays, with multiple-comparison correction as described in the Materials and Methods. Three biological replicates per group were analyzed by CyTOF at a fixed time point (day 28 after tumor implantation). Given the limited sample size, CyTOF analyses were considered exploratory. CyTOF data presented in panels B–E were analyzed using the Cytosplore-based clustering approach described above, whereas data in panel F were exported to FlowJo for downstream analysis and comparisons between groups were performed using an unpaired Student’s *t*-test. Immune populations identified by CyTOF were further evaluated in an independent experiment using multiparameter flow cytometry, including age- and sex-matched mCMV-infected, tumor-free mice (“mCMV^+^ normal brain”) and naïve, tumor-free mice as additional controls. For tumor-bearing mice, the tumor-bearing hemisphere was analyzed, whereas the corresponding hemisphere was analyzed in mCMV^+^ tumor-free mice (designated “mCMV^+^ normal brain”). Naïve mice served as negative controls for the identification and gating of mCMV-specific T cells. Flow cytometry data shown in panels G–M were analyzed using FlowJo, and comparisons between the two groups were performed using an unpaired Student’s *t*-test. The gating strategy used to identify virus-specific CD8^+^ T cells is shown in Supplementary Figure S1.

### 2.9. Flow Cytometry of TILs and Peripheral Blood

For immunophenotyping of TILs, following perfusion, the tumor-bearing right hemisphere was collected and dissociated using the Mouse Tumor Dissociation Kit (Miltenyi Biotec, Gaithersburg, MD, USA, catalog number 130-096-730) according to the manufacturer’s instructions. Leukocytes were processed immediately after isolation. For immunophenotyping of peripheral blood, samples were processed as previously described [50]. Cells were plated in non-tissue culture-treated 96-well U-bottom plates (Corning, catalog number 351177) and stained with a panel of immune cell markers. Briefly, cells were first incubated with Fc receptor blocking antibody CD16/CD32 (clone 2.4G2; BD Biosciences, Milpitas, CA, USA, catalog number 553142; 1:100), followed by staining with MHC class I mCMV tetramers M139_419–426_ -APC (1:200) and M45_985–993_ -PE (1:100), both obtained from the NIH Tetramer Core Facility. Cells were then stained with Zombie UV fixable viability dye (BioLegend, catalog number 423107; 1:1000), followed by staining with a panel of fluorophore-conjugated antibodies against surface markers (listed below). The following antibodies and reagents were used for surface staining: Zombie UV Fixable Viability Dye (BioLegend, catalog number 423107; 1:1000), CD45.2 APC-Cy7 (BioLegend, clone 104, catalog number 109823; 1:200), CD103 BV421 (BD Biosciences, clone M290, catalog number 566297; 1:100), CD8α Violet Fluor 450 (BioLegend, clone 2.43, catalog number 75-1886-U100; 1:100), PD-1 BV605 (BioLegend, clone RMP1-30, catalog number 748267; 1:200), CD3 BV711 (BioLegend, clone 17A2, catalog number 100241; 1:200), CD44 BV786 (BioLegend, clone IM7, catalog number 563736; 1:200), CD4 Spark Blue 515 (BioLegend, clone GK1.5, catalog number 100493; 1:100), NK1.1 PE-Cy7 (eBioscience, San Diego, CA, USA, clone PK136, catalog number 50-112-9610; 1:200), F4/80 APC (BioLegend, clone BM8, catalog number 123116; 1:200), Tim-3 PerCP-Cy5.5 (BioLegend, clone B8.2C12, catalog number 134011; 1:100), Gr-1 Alexa Fluor 700 (BioLegend, clone RB6-8C5, catalog number 108422; 1:800), CD62L BV650 (BioLegend, clone MEL-14, catalog number 564108; 1:200), CD11b BV510 (BioLegend, clone M1/70, catalog number 101245; 1:200), MHC class II FITC (BioLegend, clone M5/114.15.2, catalog number 107606; 1:100), CD39 PE-CF594 (BD Biosciences, clone Duha59, catalog number 143811; 1:500), and CD69 PE-Cy7 (BD Biosciences, clone H1.2F3, catalog number 552879; 1:200). After surface antibody staining, cells were washed twice with staining buffer and fixed in 100 µL fixation buffer (BD Biosciences, catalog number 554655) for 10 min at 4 °C. Cells were then washed twice, resuspended in 150 µL staining buffer, and acquired on a Cytek Aurora spectral flow cytometer (Cytek Biosciences, Fremont, CA, USA). Data was analyzed using FlowJo software v10 (Tree Star, Inc., Ashland, OR, USA).

### 2.10. Statistics

All microscope-based assays were processed and quantified using ImageJ (NIH), including particle quantification from binary images using the Analyze Particles function with automatic thresholding. Data are presented as mean ± standard deviation (SD). For comparisons between two groups, an unpaired two-tailed Student’s *t*-test was applied. For experiments involving two independent variables, two-way ANOVA was conducted with Tukey’s multiple-comparisons test. Survival data were analyzed using Kaplan–Meier survival curves, and statistical significance between groups was assessed using the log-rank (Mantel–Cox) test. All statistical analyses were conducted using Microsoft Excel 2011 (Microsoft Corp., Redmond, WA, USA) or GraphPad Prism 6 (GraphPad Software, Boston, MA, USA). A *p* value of less than 0.05 was considered statistically significant.

## 3. Results

### 3.1. Pre-Existing mCMV Infection Enhances Glioblastoma Progression in Multiple Distinct Tumor Lines

**Figure 1:**
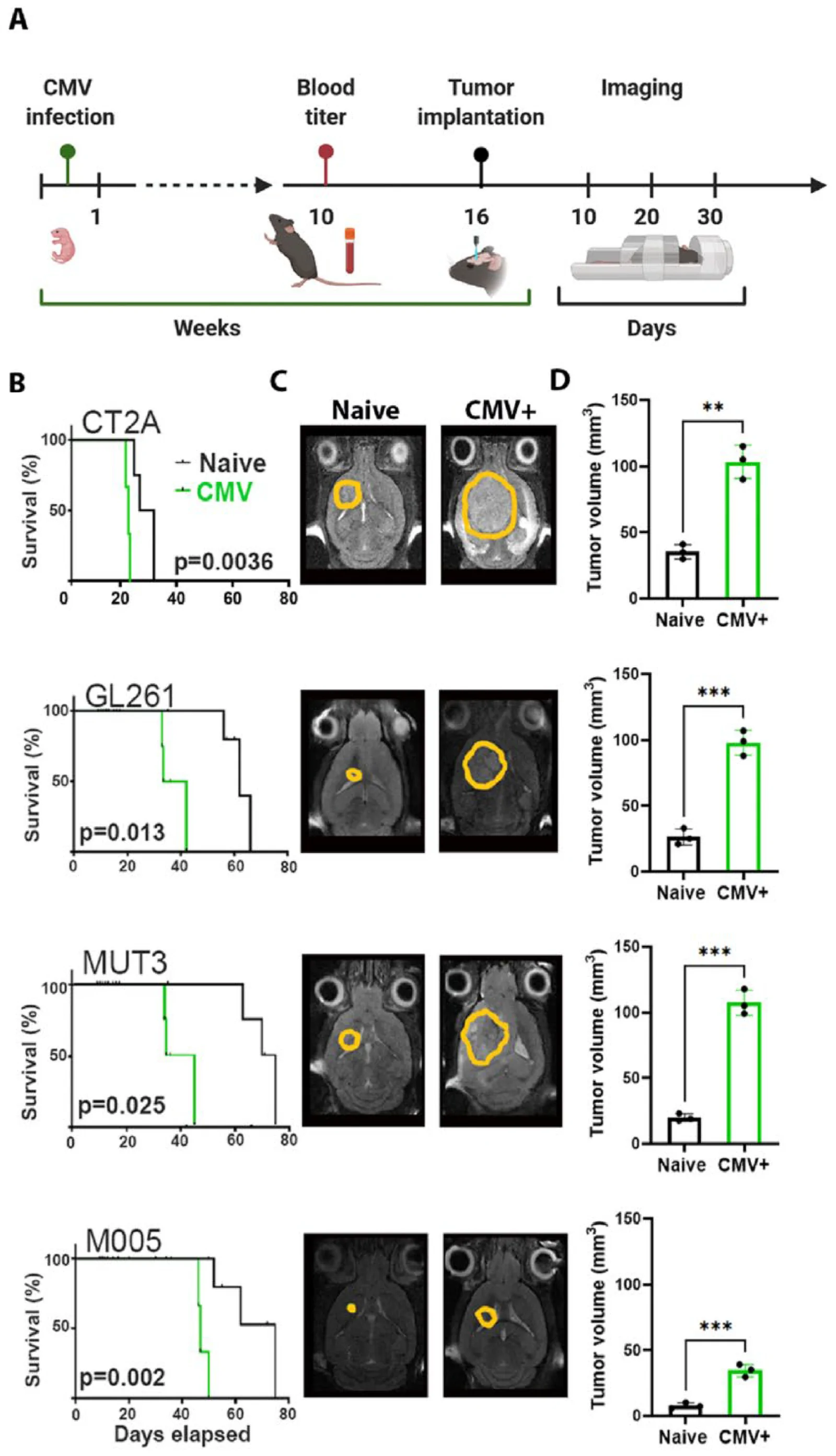
Latent mCMV is associated with tumor progression across multiple mouse models of glioblastoma. (**A**) Schematic overview of the experimental design for establishing GBM in mCMV-infected female C57BL/6 mice. (**B**) Kaplan-Meier survival curves of mice bearing orthotopic CT-2A, GL261, Mut3, or M005 tumors implanted into uninfected naïve (black) or mCMV-infected (green) mice (n = 4–5). Survival experiments were independently repeated at least twice with similar outcomes. (**C**) Representative T2-weighted MRI images of tumor-bearing brains acquired on days 10, 20 and 30 after tumor implantation, when tumor growth permitted. Representative images from day 20 for CT-2A and day 30 for GL261, Mut3 and M005 tumors are shown. (**D**) Tumor volumes quantified from MRI images on day 20 after implantation in uninfected control (n = 3) and mCMV-infected (n = 3) mice. Data are presented as mean ± SD. Statistical significance was determined using an unpaired two-tailed Student’s *t*-test (** *p* < 0.01; *** *p* < 0.001).

GBM are highly heterogeneous tumors, and we therefore sought to determine whether our previous observation that pre-existing mCMV is associated with accelerated GBM progression extends across genetically distinct tumor cell lines. We evaluated four genetically distinct murine GBM models: CT-2A (chemically induced; wild-type *Trp53*, *Pten*/*Tsc2*-deficient) [51], GL261 (chemically induced; *Kras* and *Trp53* mutations) [52], Mut3 (*Nf1* and *Trp53* inactivation) [53] and M005 (*Hras* and AKT activation in a *Trp53*+/− background) [54]. Each cell line was stereotactically implanted into female C57BL/6 mice infected at postnatal day 2 (P2) with Δm157 Smith strain mCMV (mCMV^+^) or mock-infected controls 16 weeks earlier, as previously described [45] (Figure 1A). Successful infection was confirmed by serological detection of mCMV-specific antibodies. Although tumor growth kinetics varied across models, with CT-2A tumors exhibiting the most rapid progression in mCMV^+^ mice and Mut3 and M005 tumors showing the slowest progression in mock-infected controls, all four models exhibited accelerated disease in mCMV^+^ mice. Kaplan–Meier survival analysis showed significantly reduced survival in mCMV^+^ mice across all models (Figure 1B), which is consistent with increased tumor volumes measured by T2-weighted MRI (Figure 1C,D). Together, these findings indicate that pre-existing mCMV infection is associated with accelerated GBM progression across genetically distinct murine glioblastoma models.

### 3.2. mCMV-Associated Glioblastoma Progression Is Accompanied by Histopathological Changes

**Figure 2:**
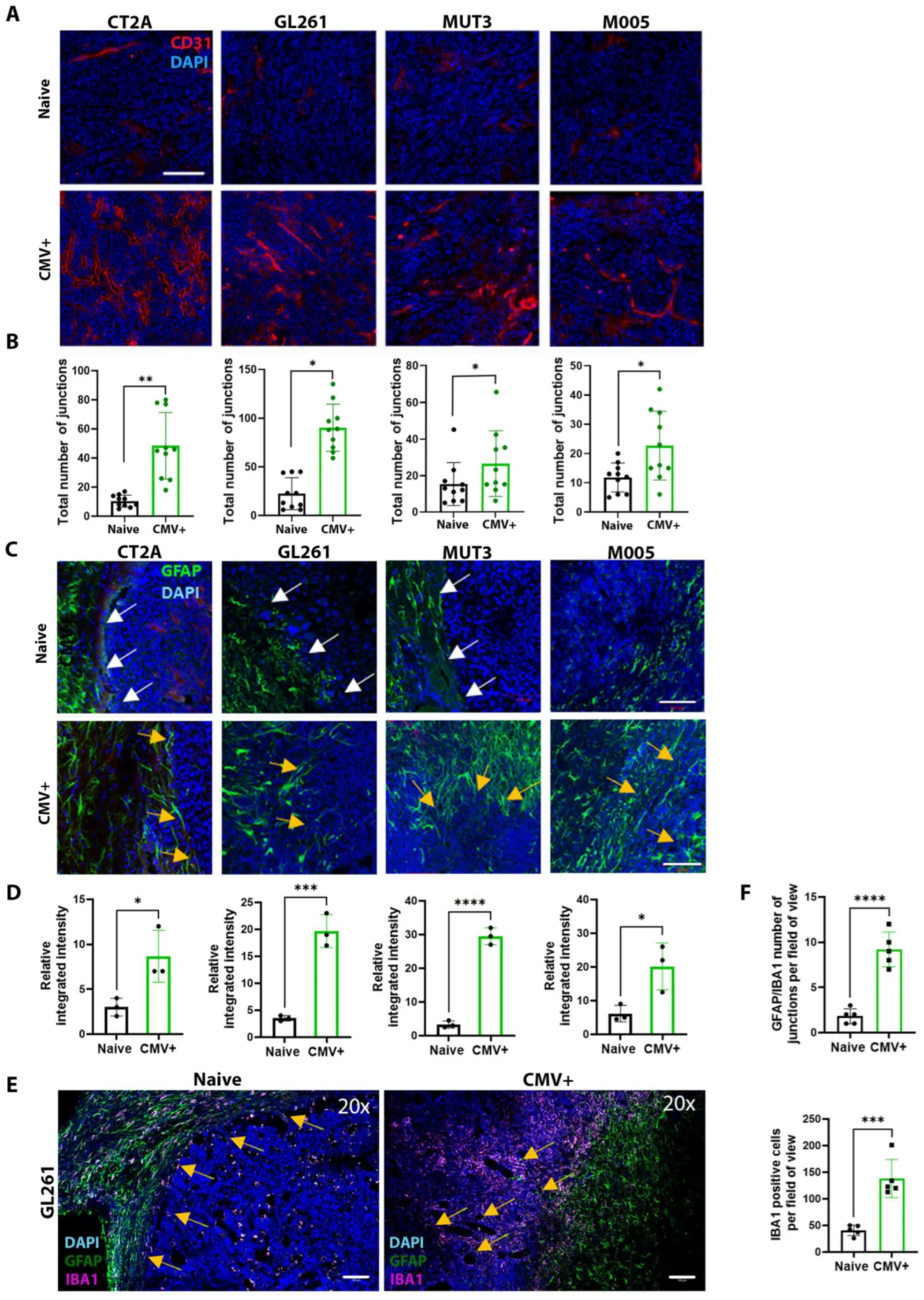
Active CMV infection is associated with increased tumor-associated blood vessels and enhanced tumor vascularization. (**A**) Representative immunofluorescence images showing CD31 staining in tumors from mCMV-infected (CMV^+^) and uninfected (naïve) mice. Scale bar, 100 μm. (**B**) Quantification of peritumoral blood vessels based on the number of vascular junctions. (**C**) Representative immunofluorescence images showing GFAP^+^ reactive astrocytes at the tumor-brain interface in mCMV-infected (CMV^+^) and uninfected (naïve) mice. Scale bar, 100 μm. (**D**) Quantification of GFAP fluorescence intensity within defined tumor regions in mCMV-infected (CMV^+^) and uninfected (naïve) mice. (**E**) Representative immunofluorescence images showing GFAP^+^ and Iba1^+^ cells and their localization in GL261 tumors (n = 5). Scale bar, 100 μm. (**F**) Quantification of GFAP^+^/Iba1^+^ cells (upper graph) and Iba1^+^ cell junctions (lower graph) per field of view in tumors from mCMV-infected (CMV^+^) and uninfected (naïve) mice. Data in panels (**A**–**F**) represent tumors from age-matched female mice collected at terminal endpoints. Data are presented as mean ± S.D. Statistical significance was determined using an unpaired two-tailed Student’s *t*-test (* *p* < 0.05; ** *p* < 0.01; *** *p* < 0.001; **** *p* < 0.0001).

Our previous work using the GL261 murine GBM model demonstrated that mCMV-associated tumor progression was accompanied by increased vascularization and poorly defined tumor margins [45]. To determine whether these features extend across genetically distinct GBM models, we analyzed tumors at day 20 after implantation. Immunohistochemical analysis of CD31 (PECAM-1), a marker of vascular endothelial cells, revealed increased tumor vascularization in mCMV^+^ mice across all four GBM models (Figure 2A). Quantification of vascular junctions confirmed a significant increase in tumor vessel density in mCMV^+^ mice compared with uninfected controls (Figure 2B). Thus, increased tumor vascularization was observed across genetically distinct models of CMV-associated GBM progression.

We next examined tumor margins by assessing astrocyte responses in mCMV^+^ and mock-infected mice. Immunofluorescence staining for glial fibrillary acidic protein (GFAP), a marker of astrocytes, revealed dense astrocyte accumulation surrounding tumors across all four GBM models (Figure 2C). Notably, the brain–tumor interfaces appeared less well defined in mCMV^+^ mice than in mock-infected controls (Figure 2C). Quantification of integrated GFAP intensity within defined tumor regions confirmed increased GFAP signal in mCMV^+^ mice (Figure 2D), consistent with increased astrocyte accumulation.

To further assess interactions between tumors and brain-associated myeloid cells, sections were stained for ionized calcium-binding adaptor molecule 1 (Iba1), a marker expressed by microglia and macrophages. Iba1 staining revealed prominent peritumoral myeloid cell accumulation in mCMV^+^ mice, which was less evident in mock-infected controls (Figure 2E). Quantification of GFAP^+^/Iba1^+^ cells and Iba1^+^ junctions between reactive myeloid cells revealed significant differences between mCMV^+^ and mock-infected mice (Figure 2E,F), consistent with altered myeloid cell accumulation and activation in the context of pre-existing mCMV infection.

### 3.3. CMV Activity and Tumor Cell Susceptibility Vary Across Murine GBM Models

**Figure 3:**
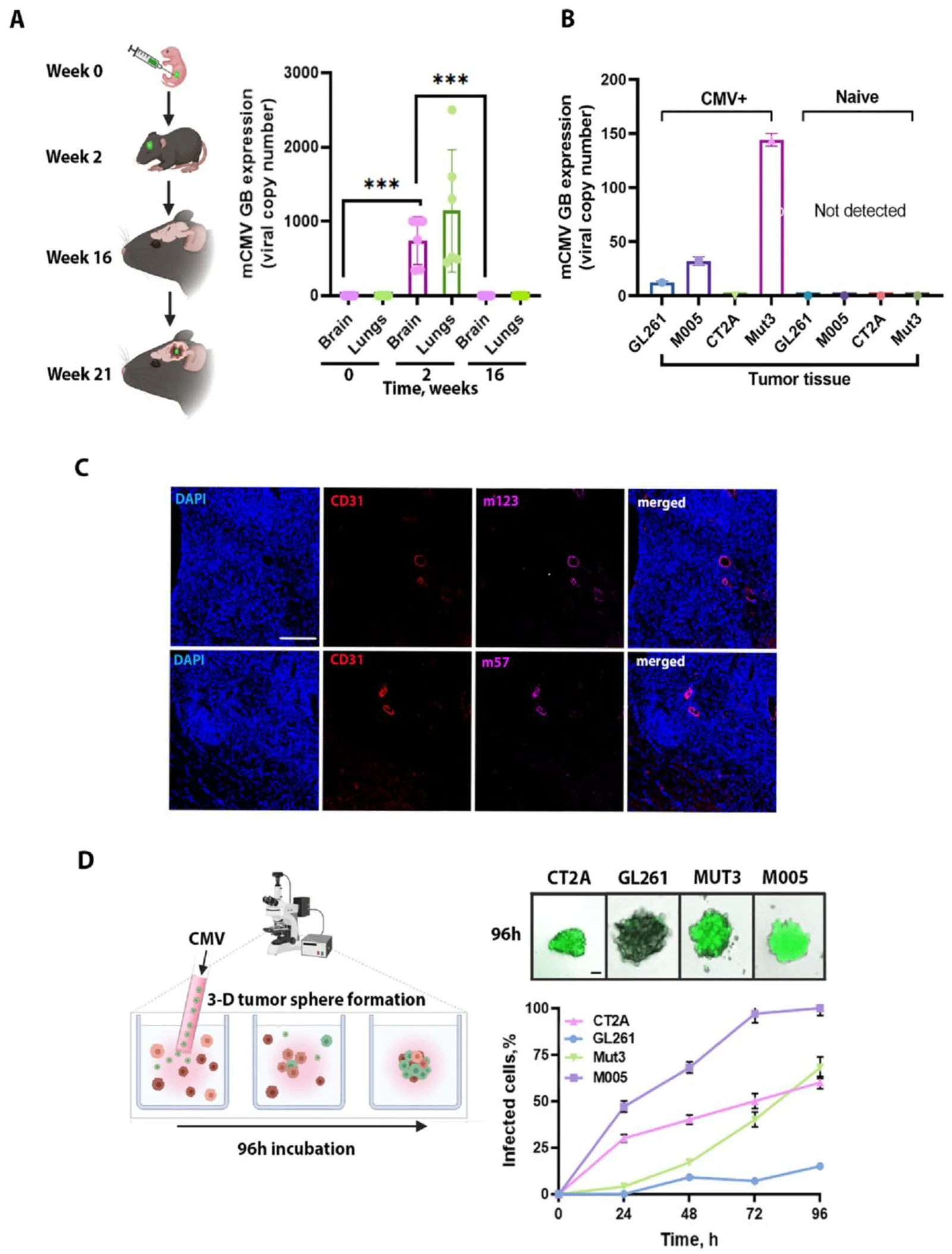
mCMV activity and infectivity in the GBM tumor microenvironment. (**A**) Schematic of the mCMV-associated GBM model and analysis of viral transcription. Bar graph shows quantitative RT-qPCR analysis of viral transcript levels in brain tissue from age-matched female C57BL/6 mice at indicated time points after mCMV infection, with lung tissue serving as a positive control (n = 6 mice/group). Data are presented as mean ± S.D. Statistical significance was determined using two-way ANOVA with Tukey’s multiple-comparisons test (*** *p* < 0.001). Non-significant differences (*p* > 0.05) are not indicated. (**B**) Quantitative RT-qPCR analysis of CMV gB transcript levels in tumor tissue at the study endpoint across four GBM models: GL261, M005, CT-2A and Mut3. Data are presented as mean ± S.D. N = 6 mice/group. (**C**) Representative immunofluorescence images showing DAPI, CD31, m123 and m57 staining and merged DAPI/CD31/m123 and DAPI/CD31/m57 channels in tumors from mCMV-infected mice. (**D**) Schematic overview of the experimental design for in vitro infectivity assays. Representative images of GFP-mCMV infection in 3D GBM neurospheres at 96 h after infection in vitro. Bar graph showing infection dynamics across the indicated GBM cell lines (n = 8 technical replicates per cell line). Data are presented as mean ± S.D. The experiment was independently repeated twice with similar outcomes.

Our previous work suggested that viral activity occurs in tumors of mice with chronic mCMV infection during the early stages after tumor implantation [45]. To define the dynamics of viral activity in these models, we measured viral transcripts by RT-qPCR in brain and lung tissues at multiple time points following infection. As shown in Figure 3A, viral transcripts were readily detectable in both brain and lungs two weeks after postnatal infection but became undetectable by 16 weeks, at the time of tumor implantation. This pattern is consistent with the establishment of chronic mCMV infection, characterized by viral persistence in the absence of readily detectable viral transcription. Tumor cells from all four GBM models were then implanted into mCMV^+^ or uninfected mice. Quantitative RT-qPCR at study endpoints detected low levels of CMV *gB* transcripts in GL261, M005 and Mut3 tumors, but not in CT-2A tumors (Figure 3B). Notably, CT-2A tumors progressed approximately twice as rapidly as the other models, resulting in earlier mortality and potentially limiting the detection of viral transcripts at the study endpoint. As viral transcripts were measured only at the endpoint, longitudinal changes in viral activity could not be assessed, and earlier or transient viral activity cannot be excluded. Immunostaining for the mCMV antigens m123 and m57 revealed sporadic viral signal, predominantly associated with blood vessels rather than tumor cells (Figure 3C). Together, these findings indicate that CMV activity is detectable within the GBM microenvironment, although its magnitude and cellular distribution vary across tumor models.

Our data demonstrate that mCMV is detectable in GL261 tumors following implantation into mice with chronic mCMV infection; however, whether the accelerated tumor growth observed in these mice is driven by viral replication within tumor cells, alterations in the tumor microenvironment, or systemic host responses remains unclear. Given that human GBM cells are susceptible to hCMV infection, we assessed the susceptibility of murine GBM models to mCMV using GFP-labeled mCMV (GFP-mCMV) to infect 3D neurosphere cultures (Figure 3D). Quantification of GFP fluorescence revealed marked differences in infection efficiency among the four cell lines. M005 neurospheres were highly susceptible, with nearly 100% of cells infected 96 h after exposure at a multiplicity of infection (MOI) of 1. Mut3 and CT-2A exhibited intermediate susceptibility, with more than 50% of cells infected at 96 h, whereas GL261 cells were comparatively resistant, with infection detected in only approximately 15% of cells (Figure 3D). Notably, in vitro susceptibility to mCMV infection did not correlate with tumor growth kinetics or overall survival in mice with pre-existing mCMV infection (Figure 1B–D), suggesting that direct tumor cell infection alone does not account for CMV-associated tumor progression in vivo.

### 3.4. Pre-Existing mCMV Infection Leads to Alterations in the Glioblastoma Tumor Microenvironment

**Figure 4:**
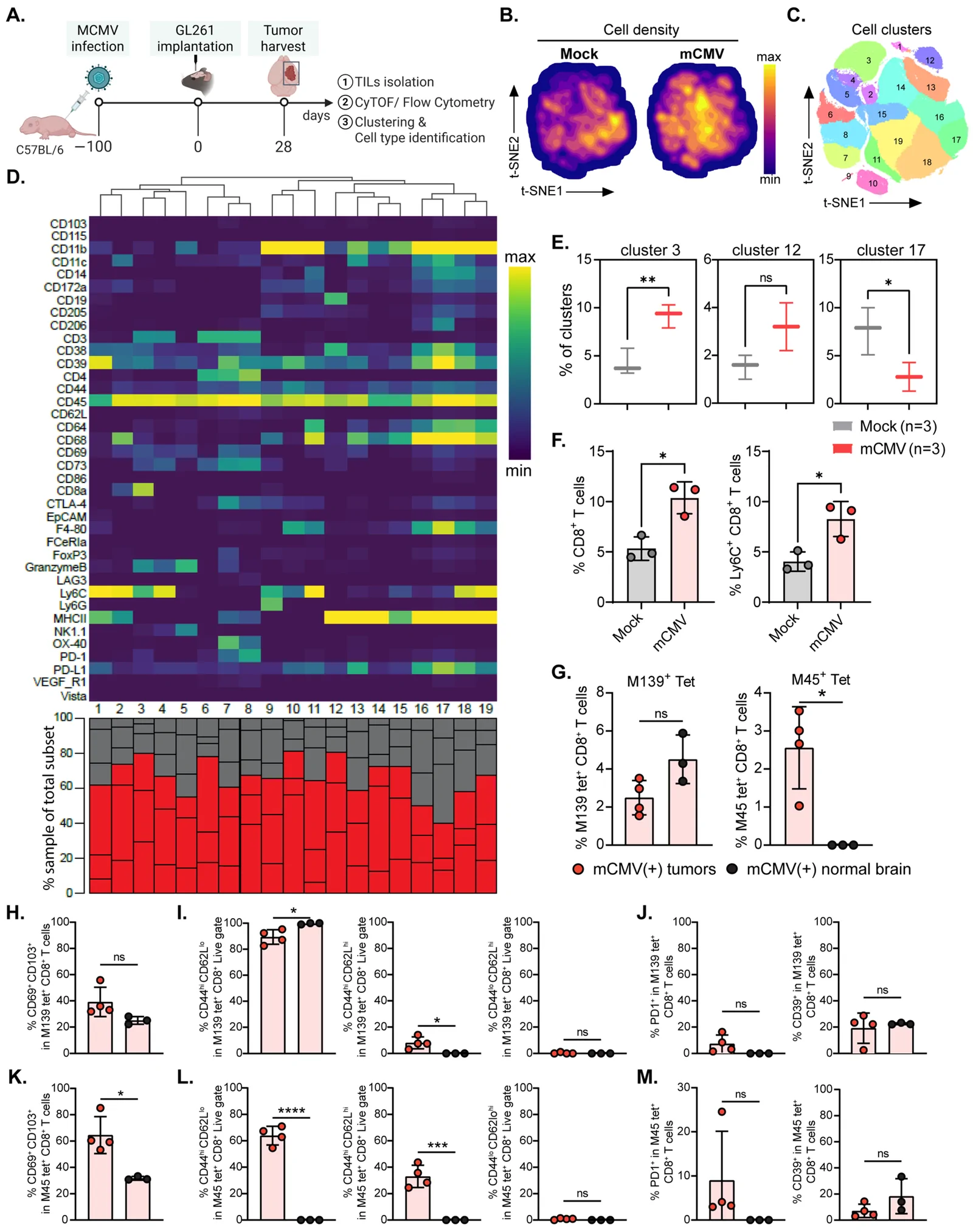
Characterization of immune infiltrates in the GBM brain microenvironment. (**A**) Experimental set up. Briefly, mice were infected with mCMV 100 days before intracranial implantation of GL261 tumor cells to establish chronic infection. On day 28 after tumor implantation, mice were perfused, and the tumor-bearing brain hemisphere was collected for downstream analysis by CyTOF and multiparameter flow cytometry. (**B**) Cell density plots showing immune cell infiltrates in the tumor-bearing hemisphere of mCMV-infected mice compared with the corresponding hemisphere of mock-infected mice bearing GL261 tumors. (**C**,**D**) Biomarker expression differences and subset percentages in mock vs. mCMV-infected groups. (**E**) Percentages of immune cell clusters 3, 12, and 17. (**F**) Percentage of CD8^+^ T cells and Ly6C^+^ CD8^+^ T cells within CD3^+^CD45.2^+^ immune cells in the tumor-bearing hemisphere of mock-infected and mCMV-infected mice bearing GL261 tumors (n = 3 mice per group). (**G**) Percentage of mCMV M139_419–426_ and M45_985–993_ epitope-specific CD8^+^ T cells in the tumor-bearing hemisphere of mCMV^+^ mice compared with the corresponding hemisphere in mCMV^+^ mice without tumors (normal brain). (**H**) Percentage of TRM (CD103^hi^, CD69^hi^) in M139_419-426_ epitope specific CD8^+^ T cells in the tumor-bearing hemisphere of mCMV^+^ mice compared with the corresponding hemisphere in mCMV^+^ mice without tumors (normal brain). (**I**) Percentages of effector memory (CD44^hi^, CD62L^lo^), central memory (CD44^hi^, CD62L^hi^) and naïve (CD44^lo^, CD62L^lo^) M139_419–426_ epitope specific CD8^+^ T cells in the tumor-bearing hemisphere of mCMV^+^ mice compared with the corresponding hemisphere in mCMV^+^ mice without tumors (normal brain). (**J**) Expression of PD1 and CD39 cell surface markers in M139_419–426_ epitope specific CD8^+^ T cells measured in the tumor-bearing hemisphere of mCMV^+^ mice compared with the corresponding hemisphere in mCMV^+^ mice without tumors (normal brain). (**K**) Percentages of TRM (CD103^hi^, CD69^hi^) M45_985-993_ epitope specific CD8^+^ T cells in the tumor-bearing hemisphere of mCMV^+^ mice compared with the corresponding hemisphere in mCMV^+^ mice without tumors (normal brain) (**L**) Percentages of effector memory (CD44^hi^, CD62L^lo^), central memory (CD44^hi^, CD62L^hi^) and naïve (CD44^lo^, CD62L^lo^) M45_985–993_ epitope specific CD8^+^ T cells measured in the tumor-bearing hemisphere of mCMV^+^ mice compared with the corresponding hemisphere in mCMV^+^ mice without tumors (normal brain). (**M**) Expression of PD1 and CD39 cell surface markers in M45_985–993_ epitope specific CD8+ T cells in the tumor-bearing hemisphere of mCMV^+^ mice compared with the corresponding hemisphere in mCMV^+^ mice without tumors (normal brain). N = 3–4 mice/group. Panels (**B**–**F**) show CyTOF data, and panels G-M show multiparameter flow cytometry data. For comparisons between two groups, data are presented as mean ± S.D. Statistical significance was determined using an unpaired two-tailed Student’s *t*-test. Statistical significance is indicated as follows: * *p* < 0.05; ** *p* < 0.01; *** *p* < 0.001; **** *p* < 0.0001. ns, not significant (*p* > 0.05).

Chronic viral infections promote clonal expansion and persistence of virus-specific immune cells within the brain [55], potentially reshaping the GBM microenvironment by either enhancing antitumor immunity or facilitating immune evasion. To determine how pre-existing mCMV infection shapes the GBM immune landscape, we used CyTOF to profile tumor-infiltrating CD45^hi^ cells in GL261-bearing mice with or without pre-existing chronic mCMV infection (Figure 4A). Tumors from mCMV^+^ mice exhibited greater heterogeneity in immune cell composition, as reflected by the broader distribution of cells across the t-SNE embedding (Figure 4B). Clustering identified 19 immune cell populations (Figure 4C and Supplementary Table S1), including an increased representation of CD8^+^ T cells (cluster 3; CD3^+^CD8α^+^Ly6C^+^Granzyme B^+^CD39^+^) and B cells (cluster 12; CD19^+^MHC II^+^CD38^+^), alongside a trend toward reduced representation of CD206^+^ macrophages (cluster 17; MHC II^+^CD11b^+^CD68^+^F4/80^+^CD64^+^CD206^+^) in the tumor-bearing hemisphere of mCMV^+^ mice (Figure 4E,F).

Multiparameter flow cytometry confirmed the presence of CD8^+^ T cells specific for inflationary (M139_419–426_) and non-inflationary (M45_985–993_) mCMV epitopes in the tumor-bearing hemisphere of mCMV^+^ mice, relative to the corresponding hemisphere of non-tumor-bearing mCMV^+^ mice (Figure 4G). A substantial fraction of these cells exhibited a tissue-resident phenotype (CD103^+^CD69^+^; Figure 4H,K) and lacked PD-1 expression (Figure 4J,M). Inflationary M139_419–426_-specific CD8^+^ T cells displayed an effector memory phenotype (CD44^hi^CD62L^lo^) in tumors and were also detectable in the brains of non-tumor-bearing mCMV^+^ mice (Figure 4I). By contrast, non-inflationary M45_985–993_-specific CD8^+^ T cells, which typically exhibit a central memory phenotype (CD44^lo^CD62L^hi^) during latent infection, displayed an effector memory phenotype (CD44^hi^CD62L^lo^) in tumors, a shift not observed in the brains of non-tumor-bearing mCMV^+^ mice (Figure 4L). Together, these findings suggest that tumor presence is associated with distinct differentiation states among mCMV-specific CD8^+^ T cells in the GBM microenvironment, although the mechanisms underlying these differences remain unclear.

To investigate how pre-existing chronic mCMV infection influences antitumor immunity, we longitudinally monitored peripheral blood immune populations in GL261 glioma-bearing mice with or without mCMV infection, from before tumor implantation through tumor progression. Immune profiling began during established viral latency (day 110 after mCMV infection) and continued until mice developed tumor-associated symptoms (day 25 after implantation), using high-parameter flow cytometry to track changes in immune cell populations over time (Supplementary Figure S2A). Before tumor implantation, mCMV-infected mice exhibited lower frequencies of circulating myeloid cells than uninfected controls (Supplementary Figure S2B). Following GL261 implantation, myeloid cells showed increased CD62L expression, consistent with changes in their migratory phenotype during tumor progression. Before tumor challenge, mCMV-infected mice also had higher frequencies of effector memory (CD44^hi^CD62L^lo^) CD8^+^ T cells with low PD-1 and CD39 expression. Following tumor implantation, CD8^+^ T-cell numbers initially became comparable between groups, but both effector and central memory subsets declined as tumors progressed (Supplementary Figure S2C). The frequencies of PD-1^+^ and LAG-3^+^ CD8^+^ T cells also decreased over time, whereas CD39 expression increased markedly in mCMV-infected mice by day 12 after implantation (Supplementary Figure S2C). CD4^+^ T-cell populations expanded similarly in both groups following tumor implantation. However, mCMV-infected mice exhibited a transient increase in CD39^+^ CD4^+^ T cells at day 12, followed by a decline coinciding with systemic lymphopenia (Supplementary Figure S2D). Tetramer analysis confirmed the persistence of M139_419–426_-specific CD8^+^ T cells before tumor implantation. These cells contracted during tumor progression, with declining frequencies of effector memory (CD44^hi^CD62L^lo^) and PD-1^+^ cells, whereas CD39 expression increased, peaking at day 12, when more than 50% of tetramer-positive cells expressed CD39 (Supplementary Figure S2E,F). Together, these findings reveal dynamic changes in systemic myeloid and T-cell compartments during GBM progression in the context of pre-existing chronic mCMV infection.

## 4. Discussion

Our findings demonstrate that chronic mCMV infection promotes GBM progression across genetically distinct glioma models, independently of tumor genotype, and is associated with substantial remodeling of the tumor microenvironment. These changes include increased tumor vascularization, alterations at the tumor–brain interface, and activation of resident myeloid cells. Comprehensive immune profiling further revealed extensive local and systemic immune remodeling, including the expansion of virus-specific CD8^+^ T cells. Collectively, these findings support a model in which chronic mCMV infection may promote GBM progression through coordinated alterations in the vascular, glial, and immune compartments, rather than through direct tumor-cell infection alone.

The relationship between CMV and GBM has remained controversial for more than two decades, with conflicting reports regarding the detection and potential biological significance of CMV in human tumor tissues [56]. Although HCMV has been proposed to contribute to GBM progression, its prevalence in human tumors and its functional relevance to disease pathogenesis remain unresolved. Extrapolation of our findings to human disease must also account for species-specific differences between mCMV and HCMV. Although mCMV and HCMV share conserved biological features, differences in viral gene content, immune evasion strategies and host interactions may influence their effects on the tumor microenvironment. Thus, whether the vascular, glial and immune alterations observed in our mCMV/GBM models are recapitulated in human GBM remains unclear. Further studies using well-characterized human tumor specimens and clinically relevant experimental models are needed to establish the translational relevance of these findings.

Despite these limitations, our results extend previous work by demonstrating a reproducible CMV-associated tumor growth phenotype across multiple GBM models with distinct genetic backgrounds. Several lines of evidence support a potential contribution of CMV activity to disease progression. Antiviral therapy has been associated with delayed progression and improved survival in subsets of patients with GBM [41,42,43], which is consistent with our previous observations in murine models [45]. CMV reactivation has also been reported in patients undergoing radiotherapy and chemotherapy, raising the possibility that treatment-associated changes in host immunity may influence viral activity and disease course [57]. In our models, low levels of CMV transcripts remained detectable in tumors following implantation, suggesting that viral activity may persist at levels below the detection limits of conventional transcriptomic approaches [58,59,60]. These observations support a role for CMV activity in disease progression while raising the question of how viral infection is translated into tumor-promoting signals within the GBM microenvironment.

Our findings argue against a simple model in which CMV accelerates GBM progression through direct infection of tumor cells. Although all GBM models tested were permissive to mCMV in vitro, their susceptibility varied substantially and did not correlate with tumor growth in vivo. Viral protein staining was predominantly localized to perivascular regions; however, the analyses performed here do not identify the cellular reservoir responsible for viral persistence or potential reactivation within the tumor microenvironment. Although this distribution suggests an association with vascular or perivascular compartments, the identity of the infected cell populations remains unresolved. Future studies should define the cellular reservoirs and functional state of mCMV within GBM and determine whether productive infection of tumor cells is required for CMV-associated tumor progression.

A prominent feature of mCMV-associated GBM progression was vascular remodeling. Across four genetically distinct GBM models, mCMV increased vascular density and branching, consistent with previous evidence linking mCMV to pro-angiogenic signaling in glioma [45]. Remodeling of the tumor–brain interface, accompanied by increased astrocyte intercalation and activation of Iba1^+^ myeloid cells, further suggests that mCMV alters the tissue architecture surrounding the tumor. These changes may be particularly relevant to GBM biology, as vascular and myeloid compartments contribute to tumor angiogenesis, invasion, and immune regulation [61]. Collectively, these findings suggest that mCMV may promote GBM progression, at least in part, by altering the cellular interactions and tissue architecture that shape the tumor microenvironment.

Chonic mCMV was also associated with extensive immune remodeling. CyTOF analysis, immunofluorescence, and peripheral blood profiling revealed changes in both tumor-associated and circulating immune populations, including an expansion of virus-specific CD8^+^ T cells. The coexistence of an enhanced antiviral response with accelerated tumor progression suggests that this response is insufficient to restrain tumor growth in the context of chronic mCMV infection. Extensive remodeling of the myeloid, vascular, glial, and stromal compartments may further constrain effective antitumor immunity, despite the increased abundance of virus-specific T cells. Whether strategies to enhance or expand virus-specific T-cell responses, including vaccination approaches targeting non-inflationary viral epitopes, could improve tumor control is an intriguing question for future investigation that remains to be experimentally tested.

An important implication of these findings is that CMV-associated effects on tumor progression cannot be predicted solely from the intrinsic susceptibility of tumor cells to infection. The lack of concordance between in vitro infection and in vivo tumor progression instead highlights the potential importance of multicellular interactions within the tumor microenvironment. This may be particularly relevant to human GBM, where genetic and cellular heterogeneity contributes to diverse vascular and immune states [62,63]. Integrating measures of CMV activity with vascular, myeloid, and immune features may therefore help identify biologically distinct GBM subsets in which CMV-associated processes represent potential therapeutic vulnerabilities.

These findings should be interpreted in light of the cohort sizes in some experiments and the exploratory nature of the CyTOF analyses, highlighting the need for further validation in future studies. Collectively, our findings support a model in which CMV promotes GBM progression through coordinated remodeling of the vascular, glial, and immune compartments of the tumor microenvironment. By shifting the focus beyond direct tumor-cell infection to the broader tissue-level consequences of CMV, this work provides a framework for investigating CMV-directed interventions alongside therapeutic strategies targeting the tumor microenvironment.

## 5. Conclusions

CMV promotes GBM progression across genetically distinct tumor models and is associated with coordinated vascular, glial, and immune remodeling. The dissociation between tumor-cell susceptibility in vitro and tumor progression in vivo, together with the predominantly perivascular localization of viral protein, implicates the tumor microenvironment as a key determinant of CMV-associated disease progression. These findings provide a framework for defining the cellular mechanisms underlying CMV-GBM interactions and for evaluating CMV-directed and microenvironment-targeted therapeutic strategies.

## Data Availability

Data presented in this manuscript are available from the corresponding authors upon reasonable request.

## Supplementary Materials

The following supporting information can be downloaded at: https://www.mdpi.com/article/10.3390/cells15211957/s1, Figure S1: Gating strategy for the identification of virus-specific CD8^+^ T cells. Representative flow cytometry plots illustrate the gating strategy used to identify mCMV-specific MHC class I tetramer-positive (tetramer^+^) CD8^+^ T cells in the brain tumor microenvironment. Cells were sequentially gated on lymphocytes (FSC versus SSC), singlets (FSC-A versus FSC-H), viable cells (7-AAD-negative) and CD45^+^ leukocytes. CD3^+^CD8^+^ T cells were then identified, with CD4^+^ cells excluded. Tetramer^+^ CD8^+^ T cells were distinguished from background staining observed in age-matched, mock-uninfected tumor-bearing mice (middle panel) and uninfected naïve mice without tumors (lower panel), with representative plots from mCMV-infected tumor-bearing mice shown in the top panel. Tetramer^+^ cells were further analyzed for CD44 and CD62L expression to distinguish effector-memory (EM), central-memory (CM) and naïve phenotypes, and for PD-1 and CD39 expression. Gates for PD-1 and CD39 positivity were defined based on the corresponding populations in uninfected naïve mice, with events exceeding the naïve control thresholds considered positive. Figure S2: GBM progression alters systemic immune responses to mCMV and reshapes systemic immunity. (A) Schematic of the experimental design for longitudinal peripheral blood immune monitoring in GL261 tumor-bearing mice. Mice were infected with mCMV 110 days before tumor implantation, and peripheral blood was collected 2 days before intracranial GL261 tumor implantation and again on days 12 and 25 after tumor implantation. Mock-infected mice served as uninfected controls, whereas mCMV-infected mice served as the infected group. (B) Percentages of CD11b^+^ myeloid cells, Gr1^+^CD11b^+^ myeloid-derived suppressor cells (MDSCs), and GL261^+^CD11b^+^ myeloid cells in the blood of mock-infected and mCMV^+^ mice at day─2 (pre-tumor), day 12, and day 25 post-tumor implantation. (C) Frequencies of effector memory (EM: CD44hiCD62Llo), central memory (CM: CD44hiCD62Lhi), and naïve (CD44loCD62Llo) CD8^+^ T cells, as well as CD69^+^, PD-1^+^, LAG-3^+^, and CD39^+^ CD8^+^ T cells in peripheral blood across the indicated time points. (D) Frequencies of EM, CM, and naïve CD4^+^ T cells, CD69^+^, CD39^+^, and LAG-3^+^ CD4^+^ T cells in peripheral blood of mock and mCMV^+^ mice. (E) Percentages of mCMV M139419-426 epitope-specific CD8^+^ T cells, including CD69^+^ and CD39^+^ subsets, at the indicated time points. (F) Memory and activation profiles (effector memory, central memory, naïve, CD69^+^, CD39^+^, PD1^+^) of M139419-426-specific CD8^+^ T cells over time. N = 6 mice per group. Data are presented as mean ± SD. Statistical significance was assessed by two-way ANOVA with Tukey’s multiple-comparisons test (* *p* < 0.05; ** *p* < 0.01; *** *p* < 0.001; **** *p* < 0.0001). Non-significant differences (*p* > 0.05) are not indicated in the graphs; Table S1: Immune cell cluster identities and defining phenotypes.
