# Supplementary material for: Cytomegalovirus Infection Is Associated with Accelerated Murine Glioblastoma Progression and Alterations in the Tumor Microenvironment
Source: Cells. 2026 Oct 28;15(21):1957. doi: 10.3390/cells15211957 (PMC13762397; doi:10.3390/cells15211957)
Supplement: Supplementary file 1 [file cells-15-01957-s001.zip › cells-4528006-supplementary.pdf]

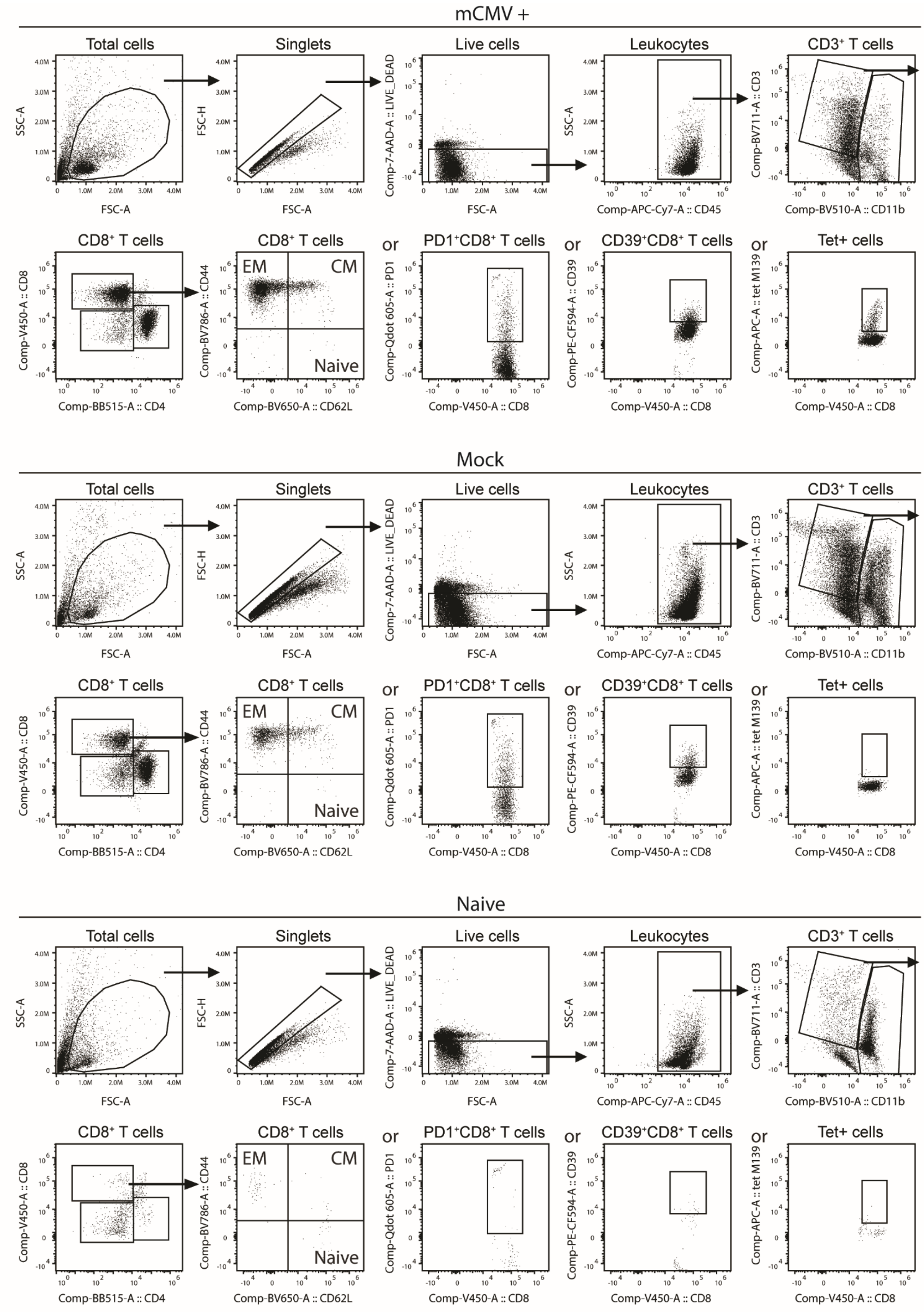
mCMV +
Total cells
Singlets
Live cells
Leukocytes
CD3⁺ T cells
SSC-A
FSC-A
FSC-H
Comp-7-AAD-A :: LIVE_DEAD
Comp-APC-Cy7-A :: CD45
Comp-BV711-A :: CD3
Comp-BV510-A :: CD11b
CD8⁺ T cells
CD8⁺ T cells
or
PD1⁺CD8⁺ T cells
or
CD39⁺CD8⁺ T cells
or
Tet+ cells
EM
CM
Naive
Comp-V450-A :: CD8
Comp-BB515-A :: CD4
Comp-BV786-A :: CD44
Comp-BV650-A :: CD62L
Comp-Qdot 605-A :: PD1
Comp-PE-CF594-A :: CD39
Comp-APC-A :: tet M139
Mock
Naive

Supplementary Figure S1. Gating strategy for the identification of virus-specific $CD8^+$ T cells. Representative flow cytometry plots illustrate the gating strategy used to identify mCMV-specific MHC class I tetramer-positive ($tetramer^+$) $CD8^+$ T cells in the brain tumor microenvironment. Cells were sequentially gated on lymphocytes (FSC versus SSC), singlets (FSC-A versus FSC-H), viable cells (7-AAD-negative) and $CD45^+$ leukocytes. $CD3^+CD8^+$ T cells were then identified, with $CD4^+$ cells excluded. $Tetramer^+$ $CD8^+$ T cells were distinguished from background staining observed in age-matched, mock-uninfected tumor-bearing mice (middle panel) and uninfected naïve mice without tumors (lower panel), with representative plots from mCMV-infected tumor-bearing mice shown in the top panel. $Tetramer^+$ cells were further analyzed for CD44 and CD62L expression to distinguish effector-memory (EM), central-memory (CM) and naïve phenotypes, and for PD-1 and CD39 expression. Gates for PD-1 and CD39 positivity were defined based on the corresponding populations in uninfected naïve mice, with events exceeding the naïve control thresholds considered positive.

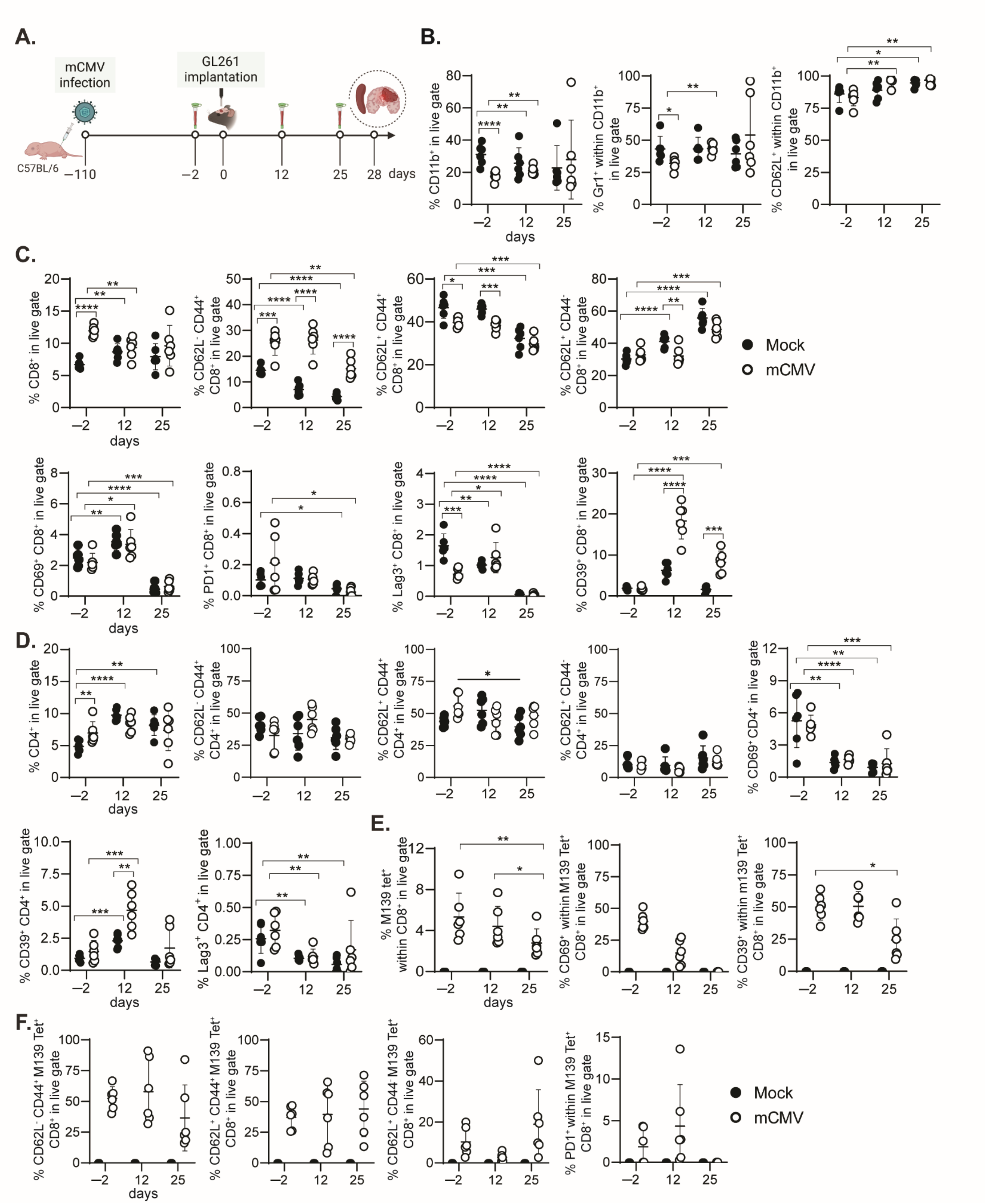
A.
mCMV infection
GL261 implantation
C57BL/6
−110
−2
0
12
25
28
days
B.
% CD11b+ in live gate
% Gr1+ within CD11b+ in live gate
% CD62L+ within CD11b+ in live gate
days
C.
% CD8+ in live gate
% CD62L- CD44+ CD8+ in live gate
% CD62L+ CD44+ CD8+ in live gate
% CD62L+ CD44- CD8+ in live gate
% CD69+ CD8+ in live gate
% PD1+ CD8+ in live gate
% Lag3+ CD8+ in live gate
% CD39+ CD8+ in live gate
Mock
mCMV
D.
% CD4+ in live gate
% CD62L- CD44+ CD4+ in live gate
% CD62L+ CD44+ CD4+ in live gate
% CD62L+ CD44- CD4+ in live gate
% CD69+ CD4+ in live gate
% CD39+ CD4+ in live gate
% Lag3+ CD4+ in live gate
E.
% M139 tet+ within CD8+ in live gate
% CD69+ within M139 Tet+ CD8+ in live gate
% CD39+ within m139 Tet+ CD8+ in live gate
F.
% CD62L- CD44+ M139 Tet+ CD8+ in live gate
% CD62L+ CD44+ M139 Tet+ CD8+ in live gate
% CD62L+ CD44- M139 Tet+ CD8+ in live gate
% PD1+ within M139 Tet+ CD8+ in live gate
Mock
mCMV
days

Supplementary Figure S2. GBM progression alters systemic immune responses to mCMV and reshapes systemic immunity. **(A)** Schematic of the experimental design for longitudinal peripheral blood immune monitoring in GL261 tumor-bearing mice. Mice were infected with mCMV 110 days before tumor implantation, and peripheral blood was collected 2 days before intracranial GL261 tumor implantation and again on days 12 and 25 after tumor implantation. Mock-infected mice served as uninfected controls, whereas mCMV-infected mice served as the infected group. (**B)** Percentages of $CD11b^+$ myeloid cells, $Gr1^+CD11b^+$ myeloid-derived suppressor cells (MDSCs), and $GL261^+CD11b^+$ myeloid cells in the blood of mock-infected and $mCMV^+$ mice at day –2 (pre-tumor), day 12, and day 25 post-tumor implantation. (**C)** Frequencies of effector memory (EM: $CD44^{hi}CD62L^{lo}$), central memory (CM: $CD44^{hi}CD62L^{hi}$), and naïve ($CD44^{lo}CD62L^{lo}$) $CD8^+$ T cells, as well as $CD69^+$, $PD\text{-}1^+$, $LAG\text{-}3^+$, and $CD39^+$ $CD8^+$ T cells in peripheral blood across the indicated time points. (**D)** Frequencies of EM, CM, and naïve $CD4^+$ T cells, $CD69^+$, $CD39^+$, and $LAG\text{-}3^+$ $CD4^+$ T cells in peripheral blood of mock and $mCMV^+$ mice. (**E)** Percentages of mCMV $M139_{419\text{-}426}$ epitope-specific $CD8^+$ T cells, including $CD69^+$ and $CD39^+$ subsets, at the indicated time points. (**F)** Memory and activation profiles (effector memory, central memory, naïve, $CD69^+$, $CD39^+$, $PD1^+$) of $M139_{419\text{-}426}$-specific $CD8^+$ T cells over time. N = 6 mice per group. Data are presented as mean ± SD. Statistical significance was assessed by two-way ANOVA with Tukey's multiple-comparisons test (*$P < 0.05$; **$P < 0.01$; ***$P < 0.001$; ****$P < 0.0001$). Non-significant differences ($P > 0.05$) are not indicated in the graphs.

Supplementary Table S1: Immune cell cluster identities and defining phenotypes.

Cluster	Proposed identity	Confidence	Defining phenotype
1	Unassigned Ly6C$^+$ MHC II$^+$ cells	Low	Ly6C$^+$ CD39$^+$ MHC II$^+$
2	Heterogeneous Ly6C$^+$ CD68$^+$ cells	Low	Ly6C$^+$ CD68$^+$ MHC II$^+$
3	CD8 T cells	High	CD3$^+$ CD8α$^+$ Ly6C$^+$ Granzyme B$^+$ CD39$^+$
4	CD4$^-$ CD8$^-$ CD3$^+$ T cells	Moderate	CD3$^+$ Ly6C$^+$
5	NK cells	Moderate	NK1.1$^+$ Granzyme B$^+$
6	CD4 T cells	High	CD3$^+$ CD4$^+$
7	Activated CD4 T cells	High	CD3$^+$ CD4$^+$ CD39$^+$ OX40$^+$ CTLA-4$^+$ CD73$^+$ PD-1$^+$
8	PD-1$^+$ CD4 T cells	High	CD3$^+$ CD4$^+$ PD-1$^+$ CD73$^+$ CD39$^+$
9	Neutrophils	High	Ly6G$^+$ CD11b$^+$ Ly6C$^+$
10	F4/80$^+$ CD11b$^+$ myeloid cells	Moderate	CD11b$^+$ F4/80$^+$
11	Ly6C-high MHC II$^-$ monocytes/macrophages	High	CD11b$^+$ Ly6C$^+$ CD68$^+$ CD64$^+$ F4/80$^+$
12	B cells	High	CD19$^+$ MHC II$^+$ CD38$^+$
13	Dendritic cells	Moderate	CD11c$^+$ MHC II$^+$ CD11b$^+$
14	CD45-low MHC II$^+$ myeloid cells	Low	MHC II$^+$ CD11b$^+$
15	CD45-low CD11b$^+$ myeloid cells	Low	CD11b$^+$ MHC II$^+$ CD68$^+$
16	MHC II$^+$ macrophages	High	MHC II$^+$ CD11b$^+$ CD68$^+$ F4/80$^+$ CD64$^+$
17	CD206$^+$ macrophages	High	MHC II$^+$ CD11b$^+$ CD68$^+$ F4/80$^+$ CD64$^+$ CD206$^+$
18	Ly6C-high macrophages	High	CD11b$^+$ MHC II$^+$ CD68$^+$ Ly6C$^+$ CD64$^+$ F4/80$^+$
19	Ly6C-high MHC II$^+$ monocytes/macrophages	Moderate	CD11b$^+$ MHC II$^+$ Ly6C$^+$ CD68$^+$ CD64$^+$

Note: Cluster identities are proposed based on the defining marker profiles; confidence levels indicate the strength of the phenotypic assignment.
